# A Study of Influence of Passengers on Driving Behavior of Drivers with Dementia or Mild Cognitive Impairment

**DOI:** 10.1002/alz.088445

**Published:** 2025-01-09

**Authors:** Mio Suzuki

**Affiliations:** ^1^ Tokai University, Hiratsuka, Kanagawa Japan

## Abstract

**Background:**

In Japan, the increase in the number of accidents involving elderly drivers has become a very serious problem. The government has implemented a cognitive function test at the time of driver’s license renewal and has taken measures to disallow driving by elderly drivers with Alzheimer’s disease, but drivers with MCI and other types of dementia continue to drive. One of the measures recommended to contribute to the safe driving of elderly drivers is to encourage them to talk to their passengers. However, for drivers with MCI who are not good at multitasking, the presence and conversation with passengers may induce dangerous behavior. Therefore, the purpose of this study is to observe the differences in cognitive function and driving behavior of elderly drivers depending on whether they have a passenger or not, and to clarify the tendency of the passenger.

**Method:**

I recorded the driving behavior of healthy elderly drivers and elderly drivers with mild cognitive impairment who live in a city in the Tokyo metropolitan area and who drive on a daily basis using a drive recorder for a month. Then, I analyzed the differences in safety confirmation between drivers with and without a passenger.

**Result:**

The results statistically showed that in Japan, where traffic is conducted on the left side of the road, the presence of a passenger increases head movement when turning left and increases eye movement alone when turning right. This means that the presence of a passenger increases safety checking behavior. In particular, the results indicate that the driver’s head moves more carefully to the left side of the road, which is difficult to look over, and that the driver’s head tends to move in the direction where the passenger is present.

**Conclusion:**

Since a passenger increases the number of safety checks during driving, it is clear that the driver may consciously try to concentrate on driving so as not to be distracted by the passenger. While driving with a passenger is expected to provide training in selecting and choosing information, it is also necessary to consider that the passenger does not interfere with the driver’s driving.

